# Axl Tyrosine Kinase Protects against Tubulo-Interstitial Apoptosis and Progression of Renal Failure in a Murine Model of Chronic Kidney Disease and Hyperphosphataemia

**DOI:** 10.1371/journal.pone.0102096

**Published:** 2014-07-14

**Authors:** Gareth D. Hyde, Rebecca F. Taylor, Nick Ashton, Samantha J. Borland, Hon Sing Geoffrey Wu, Andrew P. Gilmore, Ann E. Canfield

**Affiliations:** 1 Institute of Cardiovascular Sciences, Faculty of Medical and Human Sciences, University of Manchester, Manchester, United Kingdom; 2 Wellcome Trust Centre for Cell-Matrix Research, University of Manchester, Manchester, United Kingdom; 3 Faculty of Life Sciences, University of Manchester, Manchester, United Kingdom; UCL Institute of Child Health, United Kingdom

## Abstract

Chronic kidney disease (CKD) is defined as the progressive loss of renal function often involving glomerular, tubulo-interstitial and vascular pathology. CKD is associated with vascular calcification; the extent of which predicts morbidity and mortality. However, the molecular regulation of these events and the progression of chronic kidney disease are not fully elucidated. To investigate the function of Axl receptor tyrosine kinase in CKD we performed a sub-total nephrectomy and fed high phosphate (1%) diet to Axl+/+ and Axl−/− mice. Plasma Gas6 (Axl' ligand), renal Axl expression and downstream Akt signalling were all significantly up-regulated in Axl+/+ mice following renal mass reduction and high phosphate diet, compared to age-matched controls. Axl−/− mice had significantly enhanced uraemia, reduced bodyweight and significantly reduced survival following sub-total nephrectomy and high phosphate diet compared to Axl+/+ mice; only 45% of Axl−/− mice survived to 14 weeks post-surgery compared to 87% of Axl+/+ mice. Histological analysis of kidney remnants revealed no effect of loss of Axl on glomerular hypertrophy, calcification or renal sclerosis but identified significantly increased tubulo-interstitial apoptosis in Axl−/− mice. Vascular calcification was not induced in Axl+/+ or Axl−/− mice in the time frame we were able to examine. In conclusion, we identify the up-regulation of Gas6/Axl signalling as a protective mechanism which reduces tubulo-interstitial apoptosis and slows progression to end-stage renal failure in the murine nephrectomy and high phosphate diet model of CKD.

## Introduction

Chronic kidney disease (CKD) is a major healthcare burden and significant cause of death worldwide. Although CKD can be initiated by several different insults, it is believed to converge on common progressive pathways leading to loss of renal function and end-stage renal failure [1]. Cardiovascular disease accounts for a significant proportion of mortality within CKD patients. Indeed, vascular calcification (VC) is detected in over 80% of end-stage renal disease patients and its extent associates with increased morbidity and mortality [2], .

Axl is a member of the TAM (Tyro3, Axl and Mertk) family of receptor tyrosine kinases which are all activated by the ligand Gas6. Binding of Gas6 to Axl leads to receptor dimerisation, autophosphorylation and activation of downstream signalling pathways including PI3-Kinase/Akt and Erk1/2 resulting in pro-survival and proliferative responses, respectively [5],[6].

We have shown previously that Axl is down-regulated during phosphate-driven calcification of vascular smooth muscle cells (VSMC) and pericytes [7], [8]. Activation of Axl/PI3-kinase signalling reduces VSMC apoptosis and *in vitro* mineralisation, suggesting that Axl is protective against vascular calcification [7], [8], [9], [10]. Conversely, previous studies using Gas6−/− mice have shown a pathological role for Gas6 in nephrotoxic nephritis and streptozotocin-induced diabetic nephropathy [11], [12]. Loss of Gas6 protected against mesangial cell proliferation and glomerular hypertrophy and improved proteinuria and survival [11], [12]. These data suggest that inhibitors of the Gas6/Axl pathway may be of therapeutic benefit in these renal pathologies. However, the function of Axl in CKD and associated VC has not been investigated and whether Axl has a protective or pathological role in this setting is unknown.

Therefore, the purpose of this study was to investigate the function of Axl in CKD using a well-established murine sub-total nephrectomy and high phosphate diet model [13], [14], [15], [16], [17], [18]. We demonstrate that renal Axl and circulating Gas6 are up-regulated in Axl+/+ mice following sub-total nephrectomy and high phosphate diet. Furthermore, we show that in the absence of Axl, mice undergo rapid deterioration following sub-total nephrectomy and we uncover a novel role of Axl in protecting against tubulo-interstitial apoptosis and progression of renal failure.

## Methods and Materials

### Animal breeding and surgery

All experiments involving animals were carried out in accordance with the UK Animals (Scientific Procedures) Act 1986 (project licence number PPL 40/3518) and received local approval from the University of Manchester Ethical Review Process. All surgery was performed under isoflurane anaesthesia (4% in oxygen at 2 L/min) and with buprenorphine analgesia (0.003 mg). All efforts were made to minimize suffering.

Axl−/− mice were provided by Dr Angelillo-Scherrer (University of Lausanne, Switzerland) with permission of Prof Lemke (Salk Institute, USA) [19]. The mice had a C57/SV129 mixed background. Mice were genotyped with the following primers: Axl forward 5-TCTGGCTGGGAAAGTCAGAT-3, Axl reverse 5-CAGCCGAGGTATAGGCTGTC-3; Neo forward 5-ATGACTGGGCACAAACAGACA-3, Neo reverse 5-AATATCACGGGTAGCCAACG-3. PCR conditions were: 94°C for 10 minutes followed by 35 cycles of 94°C, 1 minute; 65°C, 1 minute; 72°C, 2 minutes and a final 10 minute incubation at 72°C. The absence of Axl in these mice was confirmed by western blotting of kidney lysates (results not shown). Initially an Axl+/− breeding pair was set up from which an Axl+/+ breeding trio (1 male, 2 females) and an Axl−/− breeding trio was derived. These 2 breeding trios produced the 23 Axl+/+ (9 males, 14 females) and 24 Axl−/− mice (10 males, 14 females) that underwent surgery in this study. Additional Axl+/+ and Axl−/− mice were bred for use as controls (no surgery, normal phosphate diet).

Sub-total nephrectomy was performed in a two-stage procedure. Mice underwent an approximate 2/3^rd^ resection of the left kidney at 11 weeks of age. This was performed by surgically removing the poles of the left kidney and applying tissue glue (Histoacryl, Braun) to curtail bleeding. One week later a complete right side nephrectomy was performed. Percentage renal mass removed was calculated by weighing the material removed in the initial partial nephrectomy, combining this with the weight of the right kidney when removed and assuming that total kidney mass was double that of the right kidney upon removal. On average 72.1% (n = 22, Std. Dev. 3.57) of renal mass was removed from Axl+/+ and 70.8% (n = 23, Std. Dev. 4.46) from Axl−/− animals, with no statistically significant difference between the groups.

Animals were placed on a 1% phosphate diet (rodent maintenance diet 1, containing 1% phosphate, Special Diet Services, UK) 1 week after the second surgery. Following surgery animals were checked daily and weighed twice per week. Animals were killed by cervical dislocation at the end of the experiment or when the predetermined humane end-point had been reached. Decisions to humanly cull animals post-surgery were based on the presence of 2 or more of the following criteria: blood present in the urine and/or faeces, weight loss of 15% or more compared to pre-surgery or 10% in the previous 72 hours, BUN greater than 100 mg/dL, piloerection and lack of response to external stimuli.

### Plasma analysis

Blood was collected by tail vein sampling during the study and by cardiac puncture at the end of the study. Blood Urea Nitrogen (BUN) levels were determined using the BUN enzymatic end point test kit (Stanbio labs, Texas, USA). Creatinine levels were determined using the mammalian serum and plasma creatinine detection kit (Arbor assays, Ann Arbor, USA). Plasma Gas6 concentrations were determined using the mouse Gas6 ELISA kit (Adipo Bioscience, Santa Clara, USA). Plasma phosphate and total calcium concentrations were determined using a Roche P8000 analyser.

### Western Blotting

Kidneys were snap frozen in liquid nitrogen, ground into a powder using a Mikro-dismembrator S (Braun Biotech International), and solubilised in lysis buffer (20 mM Tris-HCl pH 7.6, 150 mM sodium chloride, 1 mM EDTA, 1% (v/v) Igepal, 50 mM sodium fluoride, 1 mM sodium orthovanadate, 1 mM sodium pyrophosphate, and 1x protease inhibitor cocktail set 1 (Calbiochem)). Protein was quantified using the Pierce BCA protein assay kit (Thermo Scientific, UK). Samples (50 µg) were run on 10% SDS-PAGE gels and blotted with anti: Axl (1∶1000) (Santa Cruz, SC-1096, raised against a peptide mapping to the C-terminus of human Axl), phosphoAkt (1∶1000) (Cell Signalling, 9271), Akt (1∶1000) (Cell Signalling, 9272) or α-tubulin (1∶1000) (Abcam, ab4074). Membranes were incubated for 1 hour with either HRP-conjugated secondary antibodies (1∶2000) (Dako), washed, incubated with SuperSignal Chemiluminescent substrate (Pierce) and exposed to Hyperfilm (GE Healthcare) or with AlexaFluor 680-conjugated secondary antibodies (1∶5000) (Life Technologies, A21109) and visualized using an Odyssey Imaging system (LI-COR Biosciences, UK).

### Histology

Kidney remnants and thoracic aortas were washed in PBS. The adventitia was removed from aorta and tissues were fixed overnight in 4% (v/v) formaldehyde in PBS at 4°C. Samples were dehydrated and paraffin wax embedded using a microm STP 120 processor and a Microm EC 350-1/2 embedder. Seven-micron sections were cut using a Microm HM 355S and dried overnight at 37°C. All analyses were performed in a blinded manner.

Sections were stained with haematoxylin and eosin using a varistain 24-2 (Thermo-Shandon) or with picrosirius red, digitised with a 3D Histech Pannoramic 250 slide scanner and analysed using Pannoramic Viewer image analysis software. To determine the mean maximal glomeruli diameter, at least 15 glomeruli per animal were measured.

TUNEL staining was performed using the DeadEnd Fluorometric TUNEL kit (Promega, UK). TUNEL positive cells were quantified by a blinded individual (6 images per animal, 3 of the inner cortex and 3 of the outer cortex). TUNEL positive cells were expressed per unit area.

Kidney calcification was analysed by von Kossa staining. Slides were digitised with a 3D Histech Pannoramic 250 slide scanner. Calcification was quantified using HistoQuant analysis software and expressed as percentage of total surface area. VC was analysed by both von Kossa and alizarin red staining. At least 3 sections per animal were analysed. The extent of calcification for each animal was graded: 0 =  no calcification in any sections, 1 = 1 or 2 deposits in a single section, 2 = 1 or 2 deposits in multiple sections, 3 =  multiple deposits in multiple sections, 4 =  heavily calcified in multiple sections.

### Statistical Analysis

Statistical analysis was performed using GraphPad Prism. Where repeated measures of the 2 genotypes were made over time a two-way ANOVA was used to analyse the difference between genotypes followed by post hoc analysis of individual time points with Sidak compensation for multiple comparisons. Where 3 or more groups were analysed one-way ANOVA with Sidak compensation was used for parametric data and Kruskal-Wallis with Dunn' compensation for non-parametric data. Where only two groups were being compared a Student's *t*-test was used for parametric data and a Mann-Whitney test for non-parametric data. Differences in survival were determined using a Gehan-Breslow-Wilcoxon test.

## Results

### Loss of Axl results in significantly reduced survival following sub-total nephrectomy and high phosphate diet

**Figure 1:**
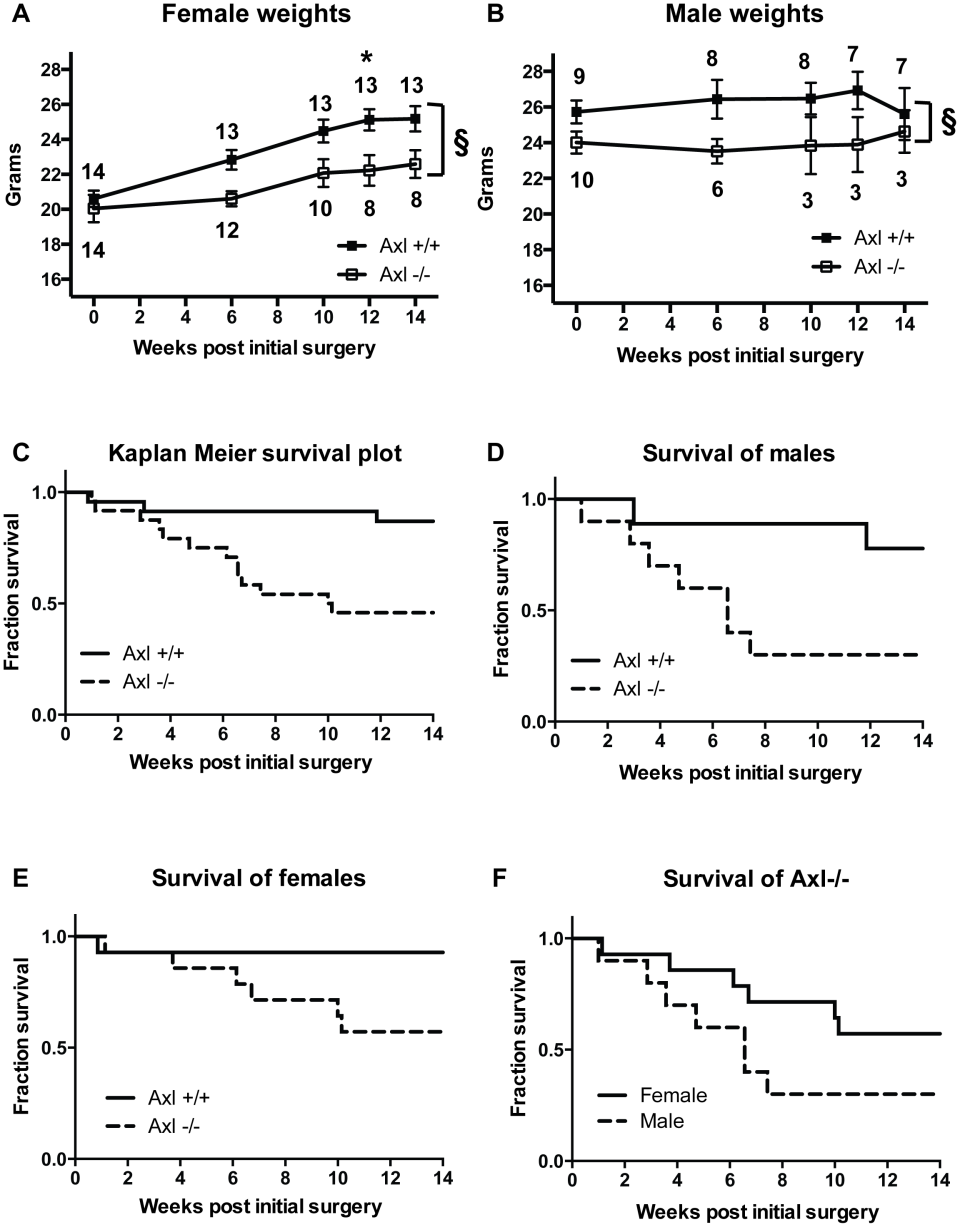
Axl−/− mice have significantly reduced body weight and survival following sub-total nephrectomy and high phosphate diet. Weight in grams of (A) females and (B) males expressed as means +/− standard error of the mean (SEM). Statistical test is a 2-way ANOVA with a Sidak compensation for multiple comparison, § = p≤0.005 for difference between genotypes and * = p≤0.05 for individual time points, n number (surviving animals) in brackets. (C–F) Kaplan-Meier survival plots of Axl+/+ and Axl−/− mice following nephrectomy and high phosphate diet. (C) All mice, (D) males, (E) females, (F) Axl−/− mice alone.

Equal amounts of renal mass were removed from both genotypes. No difference in kidney mass was observed between the genotypes either at the start of the study, upon right side nephrectomy or at the end of the study (Table 1). However, following sub-total nephrectomy and high phosphate diet Axl+/+ and Axl−/− mice exhibited marked differences in body mass (Figure 1A&B). Female Axl−/− mice did not gain weight at the same rate as Axl+/+ females, resulting in significantly lower body weights (Figure 1A). Male Axl−/− mice lost weight initially again resulting in significantly lower body weights compared to Axl+/+ controls (Figure 1B).

**Table 1:** Kidney mass pre- and post-surgery.

	Kidney mass at baseline	Percentage of kidney mass removed	Kidney remnant mass 14 weeks post-surgery
Axl+/+ females	1.47 (13, 0.18)	70.7 (13, 3.15)	0.66 (13, 0.13)
Axl−/− females	1.57 (14, 0.23)	69.9 (14, 2.55)	0.77 (8, 0.12)
Axl+/+ males	1.72 (9, 0.15)	74.0 (9, 4.40)	0.71 (7, 0.26)
Axl −/− males	1.70 (9, 0.16)	72.2 (9, 3.97)	0.70 (3, 0.09)

Kidney mass at baseline and the kidney remnant mass are expressed as a percentage of the animal's weight at these time points. When calculating kidney mass at baseline and the percentage kidney mass removed, total kidney mass was assumed to be twice that of the right kidney upon removal. The n number and standard deviation are in brackets. There is no statistically significant difference between genotypes.

Axl−/− mice also had significantly reduced survival following nephrectomy and high phosphate diet (Figure 1C). Only 45% (11/24) of Axl−/− mice survived to 14 weeks post-surgery compared with 87% (20/23) of Axl+/+ mice (p = 0.0053, hazard ratio 5.22, 95% CI 1.62–11.65). This effect was most marked in males (Figure 1D&F); only 30% of male Axl−/− mice survived to 14 weeks post-surgery, compared to 78% male Axl+/+ mice (p = 0.0370, hazard ratio 4.52, 95% CI 1.14–15.91). These mice exhibited rapid weight loss and severe uraemia (see below) and were culled before the end of the experiment having reached the predetermined humane end-point. In contrast, 57% of female Axl−/− mice survived to 14 weeks post-surgery compared to 93% of female Axl+/+ mice (p = 0.0529, hazard ratio 6.72, 95% CI 1.07–20.90) (Figure 1E&F).

### Axl−/− mice have elevated uraemia post sub-total nephrectomy and high phosphate diet

**Figure 2:**
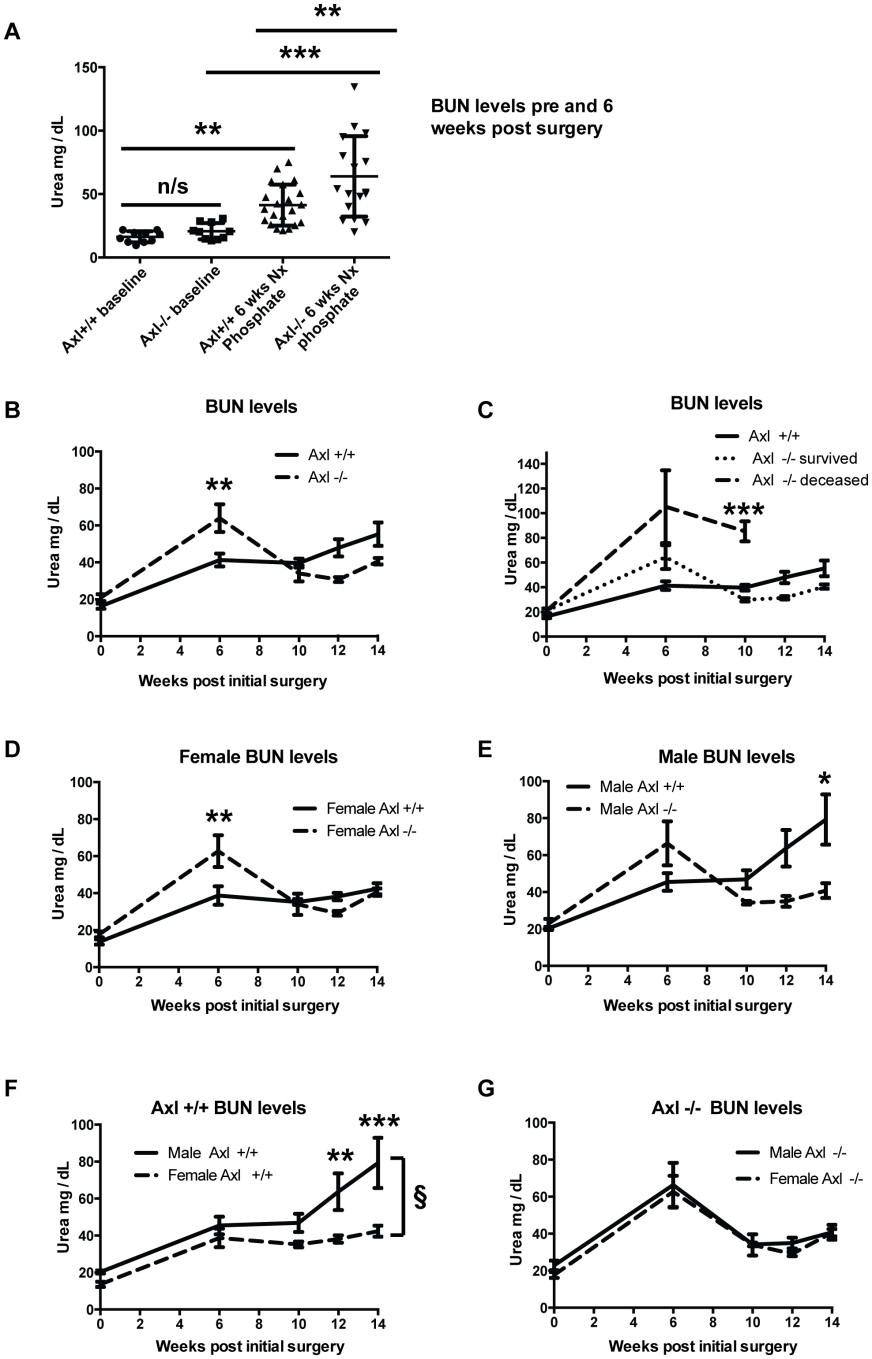
Axl−/− mice develop elevated uraemia following sub-total nephrectomy and high phosphate diet. (A) Blood Urea Nitrogen (BUN) levels expressed as individual data points with means +/− standard deviation (Std Dev). Statistical test is a 1-way ANOVA with Sidak compensation for multiple comparisons. (B–G) BUN levels of Axl+/+ and Axl−/− mice expressed as means +/− SEM; (B) all mice, (C) Axl−/− sub-populations (survived/deceased), (D) females analysed by genotype, (E) males analysed by genotype, (F) Axl+/+ mice analysed by gender, (G) Axl−/− mice analysed by gender. Statistical test is a 2-way ANOVA with Sidak compensation for multiple comparisons. § = p≤0.05 for difference between genotypes, * = p≤0.05, ** = p≤0.01, *** = p≤0.005.

As uraemia is a surrogate marker of renal function and drives VC, blood urea nitrogen (BUN) levels were monitored. In addition, plasma creatinine levels were measured at the end of the study. By 6 weeks post-surgery, BUN levels were significantly increased in Axl+/+ and Axl −/− mice (Figure 2A), confirming induction of a uraemic state. At 6 weeks, BUN levels were also significantly higher in Axl−/− compared to Axl+/+ mice (Figure 2A). Beyond 6 weeks post-surgery the BUN levels of the Axl−/− mice appeared to improve; falling below those of the Axl+/+ mice (Figure 2B). Plasma creatinine analysis 14 weeks post-surgery supported these findings (Axl+/+0.18 mg/dL, Std Dev 0.27, n = 11; Axl−/− 0.04 mg/dL, Std Dev 0.07, n = 8). The reduction in the average BUN level in the Axl−/− group is due to two reasons. Firstly, the loss of the more severely affected Axl−/− mice and their absence from later data points contributes to the reduced average BUN level in this population. By 6 weeks post-surgery, 25% of Axl−/− mice had been culled as their condition had reached the humane end-point defined in our license. Analysis confirmed severe uraemia in these mice with an average BUN concentration of 105.3±29.5 mg/dL (Figure 2C). Between 6 and 10 weeks more Axl−/− mice were lost (average BUN concentration of 85.33±8.1 mg/dL; Figure 2C). Secondly, there was an improvement in the remaining sub-population of Axl−/− mice that survived until the end of the study (Figure 2C).

Female and male Axl−/− mice showed the same trend in BUN levels (Figure 2D, E&G). However, female Axl−/− mice tolerated sub-total nephrectomy and high phosphate diet better than the Axl−/− males, and greater numbers survived to the end of the study, as shown in the Kaplan-Meier plot (Figure 1F). BUN levels were significantly higher in male Axl+/+ mice than female Axl+/+ mice 12 and 14 weeks post-nephrectomy (Figure 2F), suggesting that males progress to renal failure more quickly than females.

### Renal Axl expression is up-regulated following sub-total nephrectomy and high phosphate diet and protects against tubulo-interstitial apoptosis

**Figure 3:**
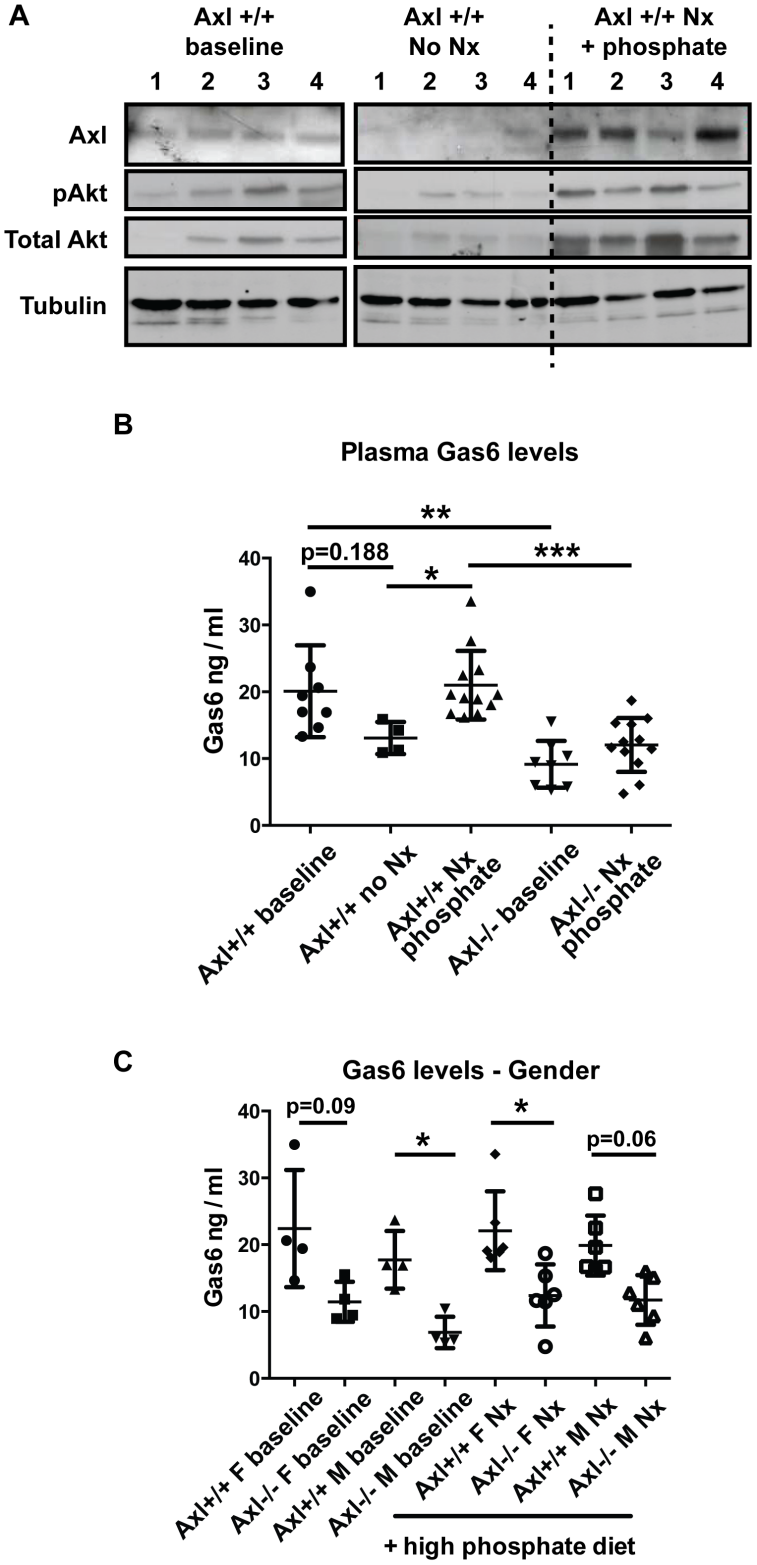
Axl and its ligand Gas6 are up-regulated following sub-total nephrectomy and high phosphate diet. (A) Western blots of kidney lysates for Axl, pAkt, total Akt and α-tubulin; 4 individual animals per group. The groups are Axl+/+ animals at baseline (11 weeks of age), 25 week old Axl+/+ animals with no nephrectomy (age-matched controls; no Nx), and 25 week old Axl+/+ animals that had a sub-total nephrectomy (Nx) at 11 weeks (Nx + phosphate). (B, C) Plasma Gas6 levels determined by ELISA; (B) Gas6 levels by genotype, (C) Gas6 levels by gender. A minimum of 4 animals were analysed per group. Results expressed as individual data points with means +/− Std Dev. Statistical test is a Kruskal-Wallis test with Dunn's compensation for multiple comparisons, * = p≤0.05, ** = p≤0.01, *** = p≤0.005.

To determine what happens to the expression of Axl and Gas6 following sub-total nephrectomy and high phosphate diet, we analysed kidney protein extracts and plasma Gas6 concentrations. Renal Axl expression was down-regulated with age in Axl+/+ mice which had not undergone surgery (11 week vs 25 week mice) (Figure 3A). However, the expression of Axl and its downstream signalling partner (Akt and pAkt) were significantly increased (6–9 fold; p<0.05) following sub-total nephrectomy and high phosphate diet compared to age-matched controls (Figure 3A). As both Akt and pAkt were upregulated the pAkt/Akt ratio did not change. Plasma Gas6 concentration also tended to decrease with age in control non-nephrectomised mice (11 vs 25 weeks); albeit not significantly. Gas6 concentrations in WT mice were significantly up-regulated following sub-total nephrectomy and high phosphate diet (Figure 3B). Furthermore circulating Gas6 concentrations were significantly reduced in Axl−/− mice, both at baseline and post-nephrectomy compared to Axl+/+ mice (Figure 3B), suggesting that Axl positively regulates the production of its ligand. Gas6 concentration did not differ between female and male Axl+/+ mice and the downward trend in Gas6 levels in Axl−/− mice was observed in both genders (Figure 3C).

To further investigate the effect of loss of Axl, sub-total nephrectomy and high phosphate diet on kidney ultrastructure, histological analyses were performed. These showed that loss of Axl had no overt effect on kidney ultrastructure at baseline (). Sub-total nephrectomy and high phosphate diet were associated with hypercellularity, tubular dilation and the recruitment of cells tentatively identified as inflammatory cells on the basis of their characteristic staining (Figure S1). However, when sections were graded for these characteristics by a blinded individual, no significant difference between genotypes was detected (data not shown).

Gas6/Axl signalling has been implicated in mesangial proliferation, glomerular hypertrophy and sclerosis [11], [12], [20], [21], [22]. We show that sub-total nephrectomy and high phosphate diet induced glomerular hypertrophy; however no significant difference between Axl+/+ and Axl−/− mice was seen (Figure S2). Sub-total nephrectomy also increased renal collagen content, but loss of Axl did not appear to modulate this effect (Figure S3).

**Figure 4.:**
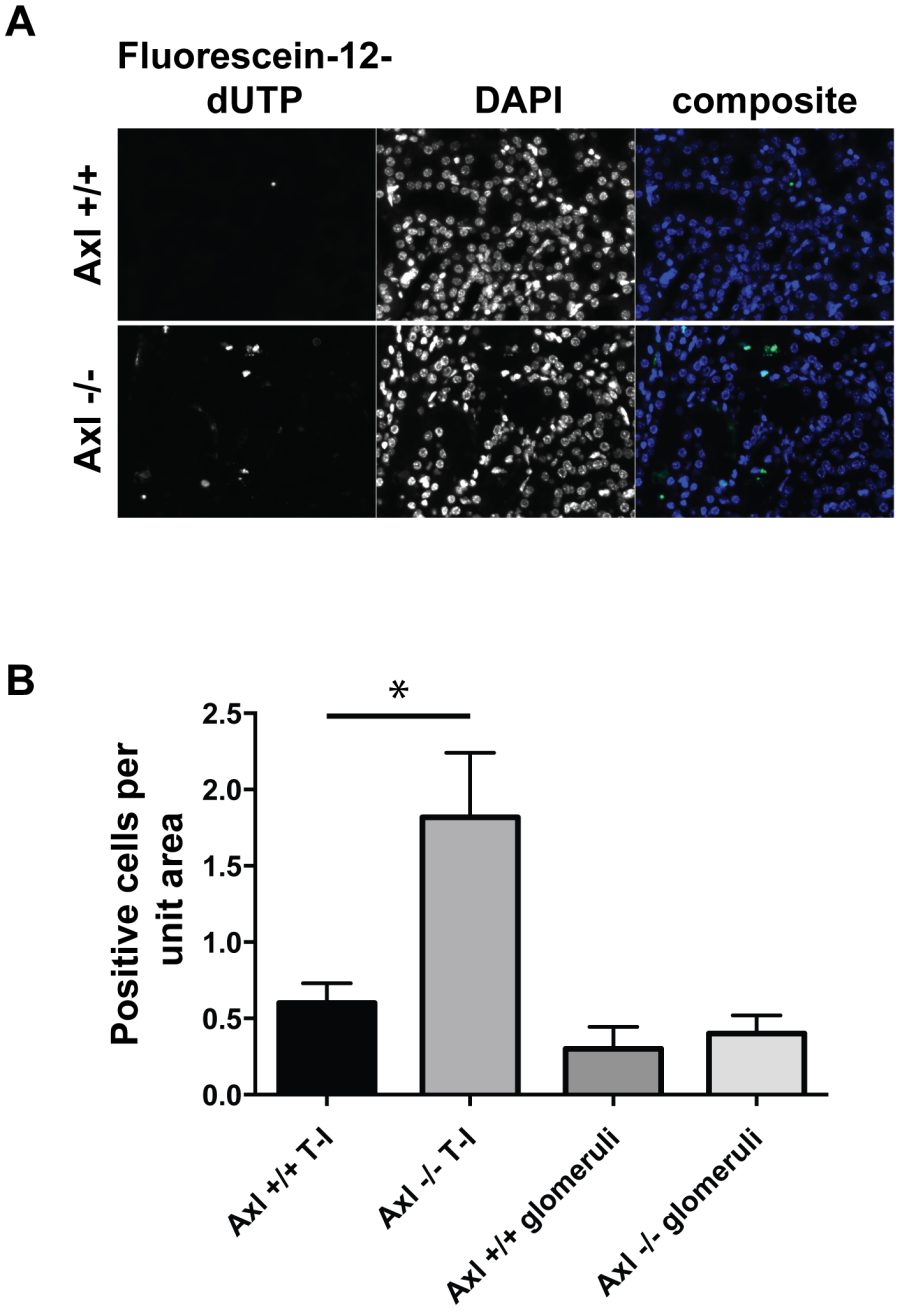
Loss of Axl results in elevated renal tubulo-interstitial apoptosis. (A) Representative TUNEL- and DAPI-stained kidney sections post-sub-total nephrectomy and high phosphate diet. Tubulo-interstitial areas of the kidney are shown, bar  = 50 microns. (B) Quantification of TUNEL staining expressed as mean +/− SEM, n = 10 per group. Statistical test is Student's *t*-test; ns  =  not significant, * = p≤0.05.

As signalling downstream of Axl commonly results in anti-apoptotic effects [5] we hypothesised that increased cell death may be occurring in the Axl−/− kidneys, contributing to the observed phenotype. We show that the number of TUNEL positive cells was significantly increased in Axl−/− kidneys compared to Axl+/+ kidneys post-nephrectomy and high phosphate diet (Figure 4A&B). No difference in TUNEL positive cells was detected within the glomeruli; however a significant increase was detected in the tubulo–interstitial regions of Axl−/− kidneys compared to Axl+/+ controls (Figure 4B).

### Sub-total nephrectomy and phosphate feeding did not induce significant vascular calcification, but increased renal calcification

Hyperphosphataemia and hypercalcaemia mediate CKD-associated VC [23]; therefore, we measured plasma phosphate and calcium concentrations at baseline and the end of the study (Table 2). Axl+/+ female mice had significantly elevated plasma phosphate and calcium concentrations 14 weeks post-surgery, as previously observed [13], [17]. Axl+/+ male mice also tended to have higher plasma phosphate and calcium concentrations, although this did not reach statistical significance. Neither female nor male Axl−/− mice exhibited hyperphosphataemia or hypercalcaemia at the end of the study.

**Table 2:** Plasma phosphate and calcium concentrations.

	Baseline, no Nx (11 weeks of age)	14 weeks post Nx + elevated Phosphate (25 weeks of age)
**Phosphate (mM)**		
Female Axl+/+	1.51+/−0.11 (5)	1.78+/−0.21 (13) *
Male Axl+/+	2.02+/−0.38 (5)	2.66+/−1.24 (7)
Female Axl−/−	1.65+/−0.37 (4)	1.69+/−0.31 (8)
Male Axl−/−	1.98+/−0.38 (8)	1.74+/−0.23 (3)
**Calcium (mM)**		
Female Axl+/+	2.02+/−0.13 (5)	2.42+/−0.29 (13) *
Male Axl+/+	2.19+/−0.37 (5)	2.51+/−0.41 (7)
Female Axl−/−	2.12+/−0.14 (4)	2.3+/−0.22 (8)
Male Axl−/−	2.34+/−0.16 (8)	2.13+/−0.06 (3)

Plasma phosphate and calcium levels in mM +/− Std Dev, n number in brackets. Statistical test is Student's *t*-test, * = p≤0.05. Nx  =  nephrectomy.

**Figure 5:**
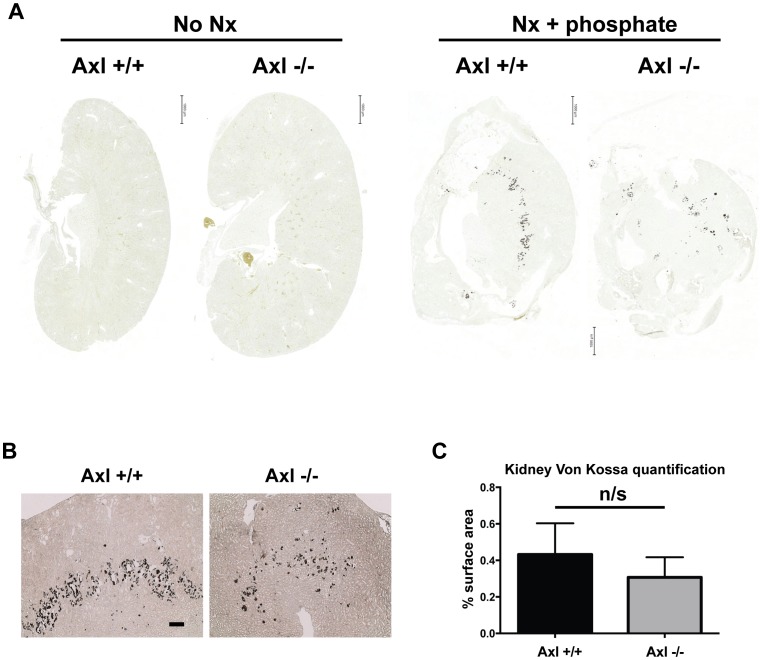
Loss of Axl did not affect the extent of renal calcification following sub-total nephrectomy and high phosphate diet. Histological analysis of kidney calcification (A) Representative scans of von Kossa stained sections from Axl+/+ and Axl−/− kidneys with and without nephrectomy and high phosphate diet. Bar  = 1000 microns. (B) Magnified images of kidney calcification 14 weeks post-initial surgery. Bar  = 200 microns. (C) Quantification of von Kossa staining, Axl+/+ n = 13, Axl−/− n = 15. Statistical test is Student's *t*-test. ns  =  not significant. Nx  =  nephrectomy.

We have shown that Axl expression is down-regulated during VSMC and pericyte mineralisation *in vitro* and activation of Axl reduces phosphate-driven mineralisation [7], [8]. Therefore, to determine whether loss of Axl affects calcification we performed von Kossa staining on kidney remnants and both von Kossa and alizarin red staining on aortic sections. The majority of kidney remnants analysed (10/13 WT and 9/15 Axl nulls) exhibited calcification post-nephrectomy and high phosphate diet. There was a downward trend in the number of animals with renal calcification and in the amount of calcification in the Axl−/− group; however histomorphometric analysis revealed no statistically significant difference between the two genotypes (Figure 5). Calcification was only detected in a minority of aorta analysed (3/10 Axl+/+ and 3/8 Axl−/− aortas); the calcium deposits were small and no significant difference was detected between the groups (results not shown). TUNEL staining did not reveal any significant induction of apoptosis in the aortas of either Axl+/+ or Axl−/− mice.

## Discussion

In this study we investigated the role of Axl tyrosine kinase in the murine sub-total nephrectomy and high phosphate diet model of CKD and VC. We demonstrate for the first time that plasma Gas6, renal Axl and downstream Akt signalling are up-regulated following sub-total nephrectomy and high phosphate diet, and that loss of Axl results in significantly elevated tubulo-interstitial apoptosis, uraemia and mortality.

Based on these results we hypothesise that the loss of Axl-dependent cell survival signalling in the tubulo-interstitium is likely to be the primary cause of accelerated renal disease and reduced survival in these animals. This is supported, first, by the fact that Axl mediates cell survival signalling in many cell types [5]. Second, tubular interstitial injury is a common mediator of CKD progression [1], [24] and finally, that we did not detect any glomerular or sclerotic defects that have previously been shown to be modified by Gas6/Axl signalling in other models of kidney disease [11], [12].

Within the kidney, Axl and Gas6 are expressed by VSMCs, glomerular mesangial cells and tubular cells in both mice and humans [22], [25], [26]. There is low expression in the basal state, but Axl and Gas6 are up-regulated in several murine models of renal disease including: Thy1.1 antibody induced glomerulonephritis, [21] nephrotoxic nephritis [12], streptozotocin-induced diabetic nephropathy [11] and a podocyte ablation model of glomerular albuminuria [26]. Furthermore, Axl and Gas6 are elevated in patients with inflammatory kidney disease [25] and IgA nephropathy [27]. Plasma Gas6 levels are also increased in patients with CKD [28]. In the disease context, Gas6/Axl signalling has been implicated mainly in cell proliferation and glomerular hypertrophy. Loss of Gas6 protected against glomerular proliferation, sclerosis and proteinuria in the nephrotoxic nephritis model [12] and reduced mesangial hypertrophy in streptozotocin-induced diabetic nephropathy [11]. Conversely, increased Gas6/Axl signalling was associated with a protective tubular proliferative response in a podocyte ablation model of glomerular albuminuria [26].

Our data together with those of other groups, suggest that Gas6/Axl signalling potentiates toxin-induced glomerular inflammatory disease but has a protective function within the tubulo-interstitium, as identified here and in the podocyte-ablation model [26]. Renal Axl expression having a protective function in kidney pathology is also consistent with a recent report examining the role of haematopoietic and non-haematopoietic Axl in DOCA salt-induced hypertension and kidney dysfunction [29]. This study demonstrated that while haematopoietic Axl had a pathological role in the model, non-haematopoietic Axl protected against renal reactive oxygen species production [29].

Gas6 can also signal via Mertk and Tyro3 which are also expressed in the kidney [30], [31]. Gas6 signalling via Mertk has been shown to be protective in the nephrotoxic nephritis model [32]. As we observed a significant reduction in plasma Gas6 concentrations in Axl−/− mice compared to Axl+/+ mice, we cannot rule out the contribution of reduced Gas6 signalling via Tyro3/Mertk to the phenotype we have characterised. It is also possible that loss of Axl modulates the expression of Tyro3/Mertk. The reduced Gas6 concentration in the Axl−/− mouse is seen in the basal state as well as the disease model. As a result, this finding has implications for all studies performed with the Axl−/− mouse when the phenotype has been attributed exclusively to reduced signalling via Axl.

Gender differences in the progression of CKD in patients [33], [34], [35] and in the rat nephrectomy model [36] have been documented previously. Here we also demonstrated clear differences between male and female nephrectomised mice. In Axl+/+ animals, sub-total nephrectomy resulted in an initial increase in uraemia which then stabilised for several weeks prior to a deterioration of only the male mice within the time frame of this study. In Axl−/− mice, both genders showed significantly elevated levels of uraemia compared to Axl+/+ mice 6 weeks post-initial surgery. However, male mice were generally unable to recover from this state, and progressed more rapidly to renal failure. Several mechanisms have been suggested to contribute to the more rapid progression of CKD in males. These include sex-dependent differences in kidney structure, glomerular hemodynamics and sex hormones. It has been proposed that estrogens have anti-apoptotic and anti-fibrotic effects on the kidney, whereas androgens promote renal apoptosis and fibrosis and thereby progression of renal disease [37], [38].

One of the objectives of this study was to investigate the role of Axl in VC associated with CKD. Surprisingly, Axl−/− mice did not demonstrate hyperphosphataemia or hypercalcaemia at the end of the study. In the absence of a full endocrine profile and a detailed assessment of renal calcium and phosphate handling, it is difficult to say why this was the case. There may have been differences in intestinal uptake; similarly renal losses of phosphate and calcium may have been greater in the Axl−/− mice. In any case, significant VC was not induced in either Axl+/+ or Axl−/− animals in the time-frame we were able to examine. We had intended to feed the high phosphate diet for a longer period in order to induce calcification; however the profound effect of the loss of Axl on renal function led to the cessation of the study after 12 weeks of phosphate feeding. The genetic background of our mice (C57/SV129 mix) may also have contributed to their reduced susceptibility to VC. Mice on this genetic background have been used for similar *in vivo* studies previously [14], although the majority of published articles use female DBA/2J mice that are particularly susceptible to calcification [13], [15], [17]. Therefore, Axl's role in VC *in vivo* remains unresolved.

Although significant vascular calcification was not induced in the model, renal calcification was induced. The calcium deposition appeared to be consistent and to occur at the cortico-medullary boundary. The conditions within the tubules of the nephrectomised mice on a high phosphate diet (e.g. low tubular fluid flow rate, high phosphate concentration) are likely to favour crystal formation, so we speculate that the calcium deposits that we observed are precursors of renal stones. However without further detailed analysis of the calcium deposits seen in the remnant kidneys, this notion remains speculation.

Axl inhibitors have received much attention in recent years, particularly in the field of oncology [39], [40]. While previous studies using Gas6−/− mice suggest that such inhibitors may be of therapeutic use in some renal pathology this article and previous work on Axl in VC [7], [8], [9], [10] indicate that such inhibitors should be studied in humans with caution, particularly in patients with renal disease.

In conclusion we have identified for the first time the up-regulation of Axl as a protective mechanism in a murine model of CKD and demonstrate that it functions to reduce tubulo-interstitial apoptosis and slow the progression of renal failure.

## Supporting Information

Figure S1 **Histological analysis of Axl+/+ and Axl−/− kidneys pre- and post- sub-total nephrectomy and high phosphate diet.** H&E stained sections of Axl+/+ and Axl−/− kidneys pre and post-nephrectomy (Nx) and high phosphate diet (14 weeks post initial surgery). Bar  = 50 microns,
(PDF)

Figure S2 **Loss of Axl does not modulate glomerular hypertrophy post sub-total nephrectomy and high phosphate diet.** Representative H&E stained glomerular sections. Bar  = 20 microns. Arrow indicates how maximal diameter of glomeruli was measured. (B) Quantification of mean maximal glomerular diameter. Axl+/+ n = 4, Axl −/− n = 3, Axl+/+ Nx n = 13, Axl −/− n = 15. Results expressed as means +/− SEM. Statistical test is a Kruskal-Wallis test with Dunn' compensation for multiple comparisons, * = p≤0.05.
(PDF)

Figure S3 **Loss of Axl does not modulate renal collagen content post sub-total nephrectomy and high phosphate diet.** (A) Representative images of picrosirius red stained kidney sections. Bar  = 100 microns. (B) Frequency of positive picrosirius red staining in Axl+/+ and Axl−/− kidney post sub-total nephrectomy.
(PDF)
