# Supplementary figures and images for: Axl Tyrosine Kinase Protects against Tubulo-Interstitial Apoptosis and Progression of Renal Failure in a Murine Model of Chronic Kidney Disease and Hyperphosphataemia
Source: PLoS One. 2014 Jul 14;9(7):e102096. doi: 10.1371/journal.pone.0102096 (PMC4096921; doi:10.1371/journal.pone.0102096)

Figure S1

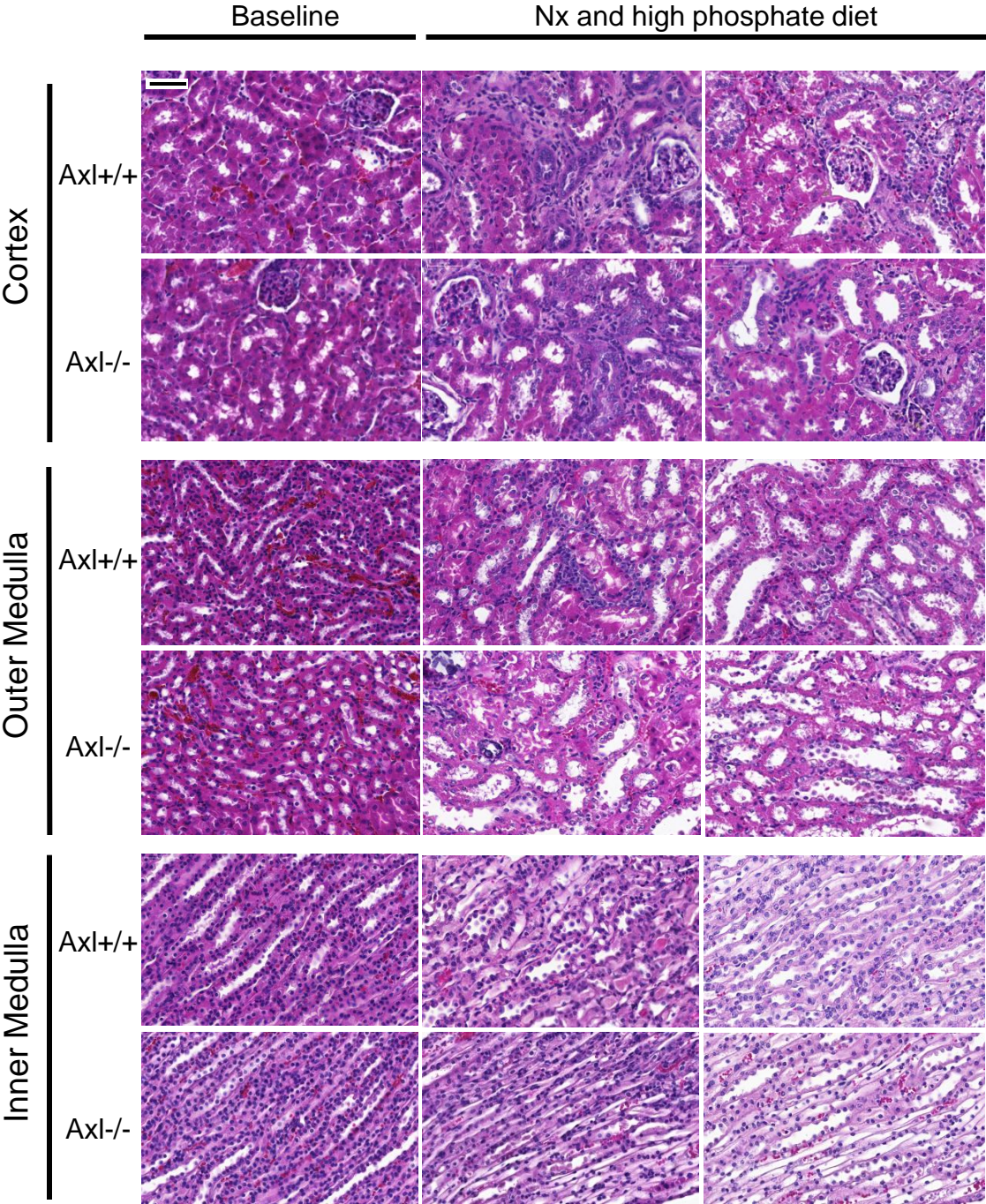

Supplement: Figure S1 — Histological analysis of Axl+/+ and Axl−/− kidneys pre- and post- sub-total nephrectomy and high phosphate diet. H&E stained sections of Axl+/+ and Axl−/− kidneys pre and post-nephrectomy (Nx) and high phosphate diet (14 weeks post initial surgery). Bar = 50 microns, (PDF) [file pone.0102096.s001.pdf]

Figure S2

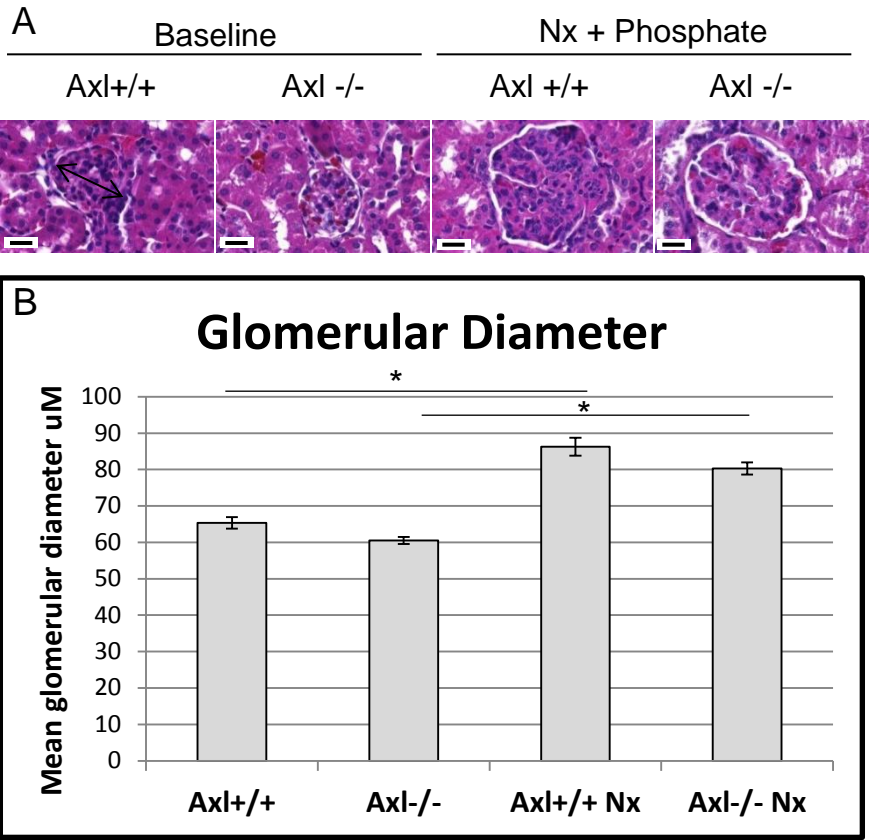

Supplement: Figure S2 — Loss of Axl does not modulate glomerular hypertrophy post sub-total nephrectomy and high phosphate diet. Representative H&E stained glomerular sections. Bar = 20 microns. Arrow indicates how maximal diameter of glomeruli was measured. (B) Quantification of mean maximal glomerular diameter. Axl+/+ n = 4, Axl −/− n = 3, Axl+/+ Nx n = 13, Axl −/− n = 15. Results expressed as means +/− SEM. Statistical test is a Kruskal-Wallis test with Dunn' compensation for multiple comparisons, * = p≤0.05. (PDF) [file pone.0102096.s002.pdf]

Figure S3

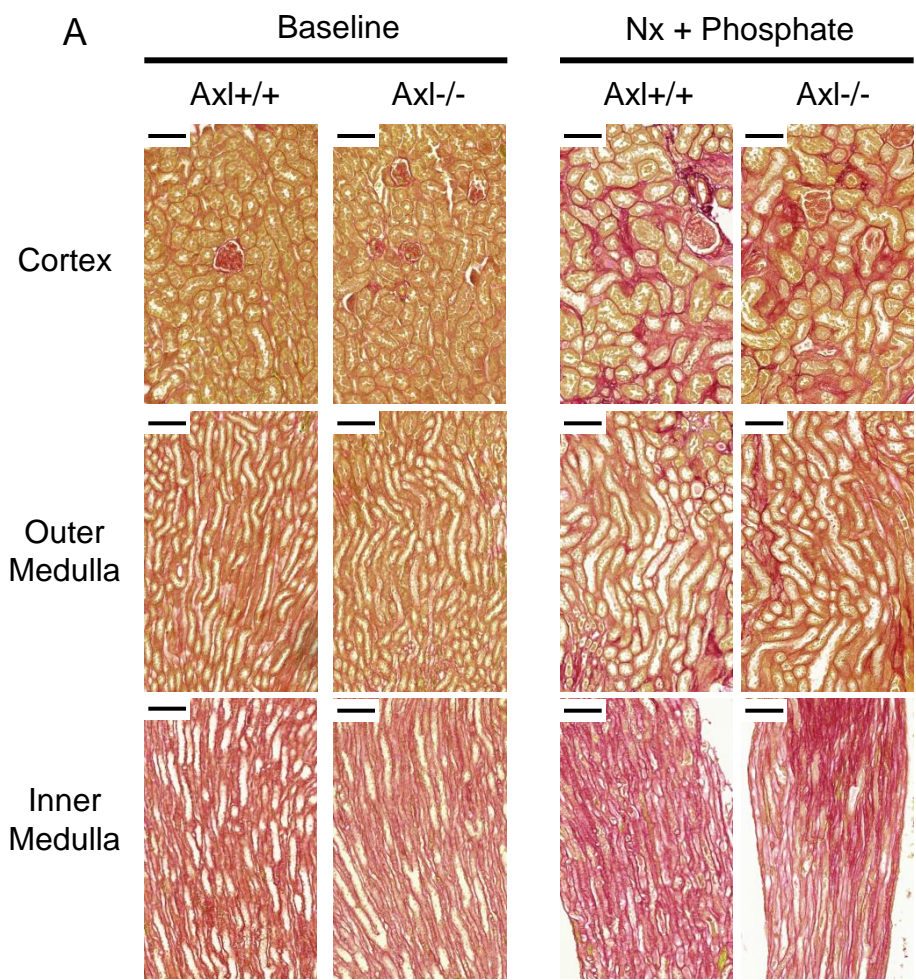

**B**

|        | Cortex     | Outer medulla | Inner medulla |
|--------|------------|---------------|---------------|
| Axl+/+ | 46% (6/13) | 85% (11/13)   | 62% (5/8)     |
| Axl-/- | 60% (9/15) | 73% (11/15)   | 66% (4/6)     |

Supplement: Figure S3 — Loss of Axl does not modulate renal collagen content post sub-total nephrectomy and high phosphate diet. (A) Representative images of picrosirius red stained kidney sections. Bar = 100 microns. (B) Frequency of positive picrosirius red staining in Axl+/+ and Axl−/− kidney post sub-total nephrectomy. (PDF) [file pone.0102096.s003.pdf]
